# Dynamic Properties and Fractal Characteristics of 3D Printed Cement Mortar in SHPB Test

**DOI:** 10.3390/ma14195554

**Published:** 2021-09-24

**Authors:** Yixin Mo, Songlin Yue, Qizhen Zhou, Bowei Feng, Xiao Liu

**Affiliations:** College of Defense Engineering, Army Engineering University of PLA, Nanjing 210007, China; myx91@foxmail.com (Y.M.); zhouqizhen2016@163.com (Q.Z.); fengbwacy@126.com (B.F.); xiao_Liu_z@163.com (X.L.)

**Keywords:** 3D printing, mortar, dynamic compressive strength, anisotropy, energy dissipation density, fractal dimension

## Abstract

Comparing with the traditional construction process, 3D printing technology used in construction offers many advantages due to the elimination of formwork. Currently, 3D printing technology used in the construction field is widely studied, however, limited studies are available on the dynamic properties of 3D printed materials. In this study, the effects of sand to binder ratios and printing directions on the fractal characteristics, dynamic compressive strength, and energy dissipation density of 3D printed cement mortar (3DPCM) are explored. The experiment results indicate that the printing direction has a more significant influence on the fractal dimension compared with the sand to binder ratio (S/B). The increasing S/B first causes an increase and then results in a decline in the dynamic compressive strength and energy dissipation of different printing directions. The anisotropic coefficient of 3DPCM first is decreased by 20.67%, then is increased by 10.56% as the S/B increases from 0.8 to 1.4, showing that the anisotropy is first mitigated, then increased. For the same case of S/B, the dynamic compressive strength and energy dissipation are strongly dependent on the printing direction, which are the largest printing in the Y-direction and the smallest printing in the X-direction. Moreover, the fractal dimension has certain relationships with the dynamic compressive strength and energy dissipation density. When the fractal dimension changes from 2.0 to 2.4, it shows a quadratic relationship with the dynamic compressive strength and a logarithmic relationship with the energy dissipation density in different printing directions. Finally, the printing mortar with an S/B = 1.1 is proved to have the best dynamic properties, and is selected for the 3D printing of the designed field barrack model.

## 1. Introduction

With the development of the global construction industry, traditional construction is facing the problems of environmental pollution, a shortage of labor, overuse of raw materials, etc. [1,2]. Compared with the traditional construction process, 3D printing technology, as used in construction, has the advantages of high design flexibility, short construction periods for the single irregularly shaped components, and lower resource consumption due to the elimination of formwork [3,4,5]. These advantages of 3D printing techniques can resolve a series of problems faced by traditional construction [6]. Hence, with the development of building digitization and automation, 3D printing technology is being studied extensively in civil engineering [7,8].

In recent years, many buildings were successfully built with the aid of the rapid development of 3D printing techniques [9,10,11]. For those printed buildings with cementitious materials, it is not required to have a beautiful appearance, but they must have enough strength to ensure their safety in use. As a result of the special extrusion process and layer-by-layer stacking, the overall strength of printed cementitious materials is greatly influenced by the interlayer bond strength, so the research tends to focus on the interlayer bond strength (Table 1) [12]. Moreover, the fundamental mechanical behaviors of 3D printed cementitious materials are also dependent on printing directions, giving rise to the anisotropy of mechanical properties. In the experimental results of Pham et al. [13], the compressive strengths of the printing direction and the horizontal expanding direction were lower than that in the vertical build-up direction. Similar results were also found in terms of the tensile, bending and shearing strength [14].

The properties of materials under dynamic loading change greatly due to the strain rate effect, which is different from that under static loading. Up to now, the dynamic properties of many cementitious materials have been investigated through the Split Hopkinson Pressure Bar (SHPB) test (Table 2); however, limited studies are available on the dynamic properties of 3D printed cementitious materials using a SHPB. In defense engineering, buildings are usually designed to bear dynamic loading [18]. The US Marine Corps used 3D printing to build a concrete barrack within 40 h, and the printed living space could withstand a certain impact and explosion [19]. In addition, defense works and civil infrastructure are at risk of terrorist attack. In consequence, the dynamic mechanical behaviors of printed cementitious materials warrant investigation.

Since fractal characteristics exist in the evolution of structure from original defects to macroscopic fractures, many research studies on the fracture behavior of materials have adopted fractal geometry theory [23,24]. Energy dissipation analysis is considered as an important index to reveal the fracturing mechanism of materials, which has been widely applied to the research on the impact crushing characteristics of materials, such as rock [25], concrete [26], and porous materials [27]. The application of fractal theory and energy dissipation analysis can provide a theoretical foundation for analyzing the fractal characteristics of fragments [28]. However, there are relatively few studies of the fractal feature and energy dissipation characteristics of 3D printed cementitious materials.

A 100-mm-diameter SHPB was adopted to measure the dynamic behavior of 3D printed cement mortar (3DPCM). The purpose of this work is to investigate the effects of sand to binder ratio (S/B) and printing direction on the fractal features and dynamic behaviors of 3DPCM. For the 3DPCM with different sand to binder ratios, the fractal characteristics in different printing directions were quantitatively studied by the fractal dimension. In addition, the anisotropy coefficients with different sand to binder ratios were compared. The compactness of 3DPCM in different printing directions was evaluated by use of the ultrasonic pulse velocity (UPV–NU61, Tiance Technology Company, Nanjing, China). The corresponding empirical equations were obtained by correlating dynamic compressive strength and energy dissipation density with the fractal dimension, respectively. The printing mortar with S/B = 1.1 was selected to print a full-scale model barracks. The findings provide a certain reference for understanding the dynamic performance of 3D printed cementitious materials.

## 2. Experimental Process

### 2.1. Preparation of Printable Mortar

This work attempts to develop a printable 3D printing cement mortar based on S/B of 0.5 to 1.7 (0.5, 0.8, 1.1, 1.4, and 1.7). The binder component used in this experiment consists of ordinary Portland cement (Grade 42.5;), fly ash 33% and silica fume 2% (by mass of cement). The water to cement ratio of the prepared cement mortar is about 0.34. To improve the fluidity and viscosity, polycarboxylate superplasticizer 0.1% and thickening agent 0.05% (by mass of cement) were added to the mixture, respectively. Table 3 displays the particle size distribution of the river sand used in the present work.

**Figure 1:**
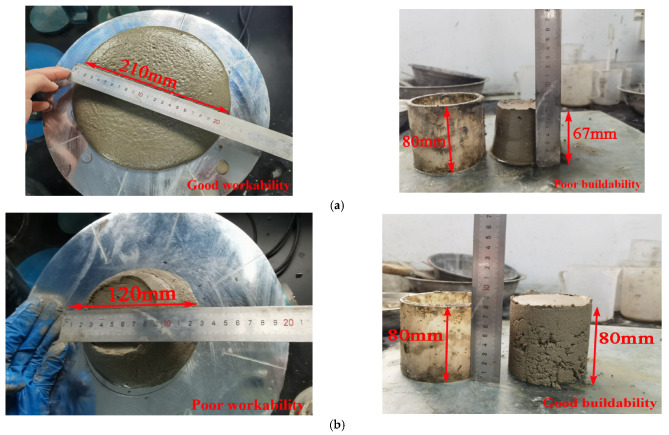
Printability tests with (**a**) S/B = 0.5 and (**b**) S/B = 1.7.

According to the convenient method for evaluating the changes in workability and buildability with time intervals [29], as illustrated in Figure 1, mortar specimens prepared with S/B = 0.5 and S/B = 1.7 fail to satisfy the requirement of printability due to the poor buildability and workability, respectively. Based on trial-and-error optimization, when 0.8 ≤ S/B ≤ 1.4, the prepared mortar could be used for printing and mortar mixes with S/B = 0.8, 1.1, and 1.4 were selected to evaluate the dynamic properties of 3DPCM. The printability test results of the mortar with three different sand to binder ratios are summarized in Table 4.

### 2.2. 3D Printing Process and Specimen Processing

**Figure 2:**
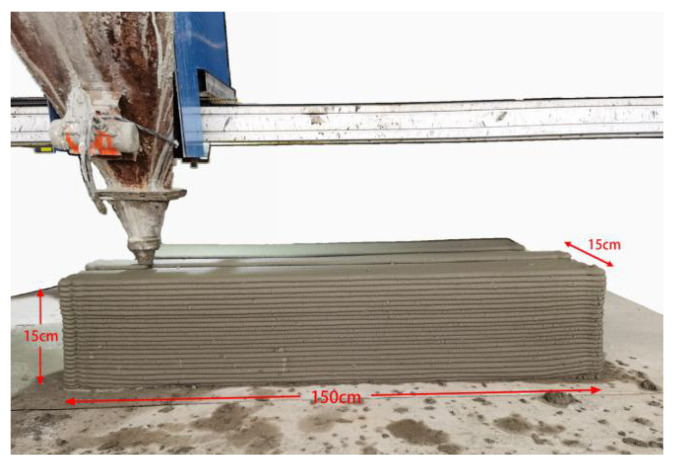
3D printed cuboid.

**Figure 3:**
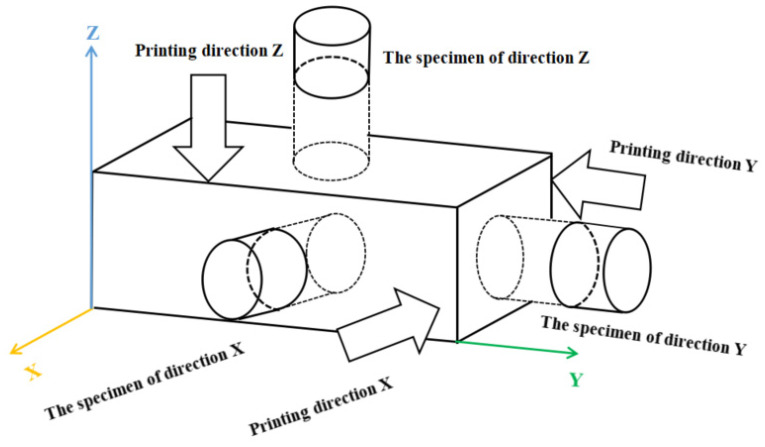
The sampling process.

A large-scale Cartesian frame 3D printer with a mobile platform along the X, Y, and Z-axes was used: The printer has a round nozzle with a diameter of 20 mm, and the effective printing space is 3 m (L) × 3 m (W) × 3 m (H). During the printing process, the fresh mixture was spirally squeezed from the V-shaped storage bin to the nozzle, and printed by the nozzle at a horizontal speed of 9 m/min. The size of the extruded filament was about 22–25 mm in width and 13–15 mm high. The printing mortar was finally printed into cuboids measuring 1500 mm (L) × 150 mm (W) × 150 mm (H) (Figure 2). Each printed cuboid was cured at 20 °C and a relative humidity of 90% for 28 d. After the curing period, a certain number of cylinder specimens were processed from the three printing directions (X, Y, Z) of the printed cuboids, which had a diameter of 80 mm (D) and a length of 40 mm (L), as shown in Figure 3. Furthermore, some cast cylinder specimens with the same size and mix proportions as the printed cylinder specimens were prepared for comparisons.

### 2.3. UPV Testing

Due to the special stripe stacking characteristics of 3D printing technology, a certain number of voids and flaws in the interlayers between filaments can be formed during the deposition process. When ultrasonic waves transmit in the printed specimen, the voids and flaws inside printed specimens may change the form of ultrasonic waves through absorbing and reflecting waves, thus affecting the wave energy and transmission time [30]. The more defects, the lower the density, and UPV [31]. Therefore, it is reasonable to reflect the void features of 3DPCM in different printing directions based on the variation of UPV.

The velocity *v* is calculated from the thickness of specimen *P* and the transmission time *T,* as follows:

(1) $$v=\frac{P}{T}$$

**Figure 4:**
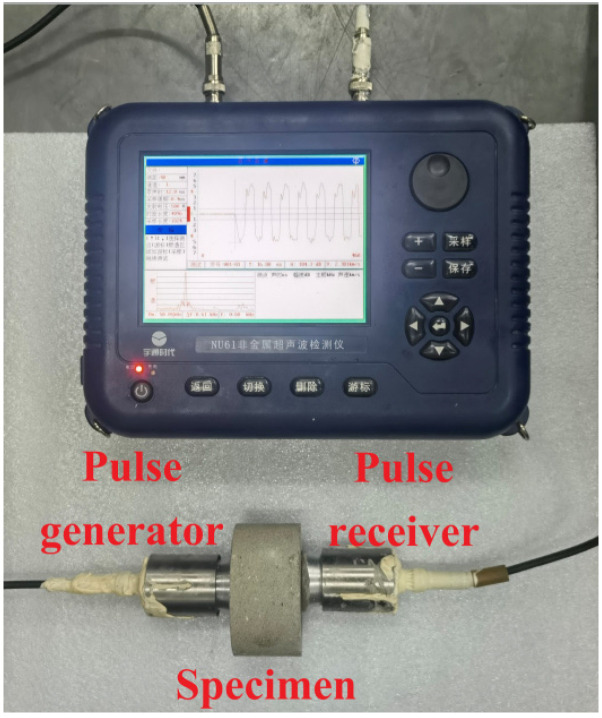
The UPV testing of specimens.

The UPV testing of specimens processed from three printing directions (X, Y, Z) was conducted at a pulse frequency of 60 kHz. The surfaces of two transducers and specimen were smeared with a small amount of Vaseline to ensure good contact between the transducer and the specimen (Figure 4). Three measuring points in the outer surfaces of the specimen were selected for testing and the average value of measurement results was regarded as the UPV value of 3DPCM in this direction.

### 2.4. Dynamic Compression Test

**Figure 5:**
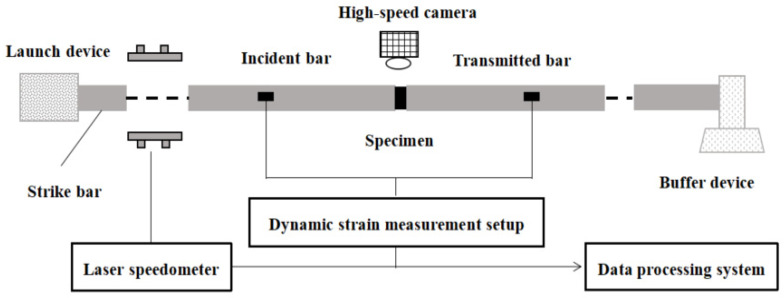
The set-up of 100 mm-diameter SHPB.

**Figure 6:**
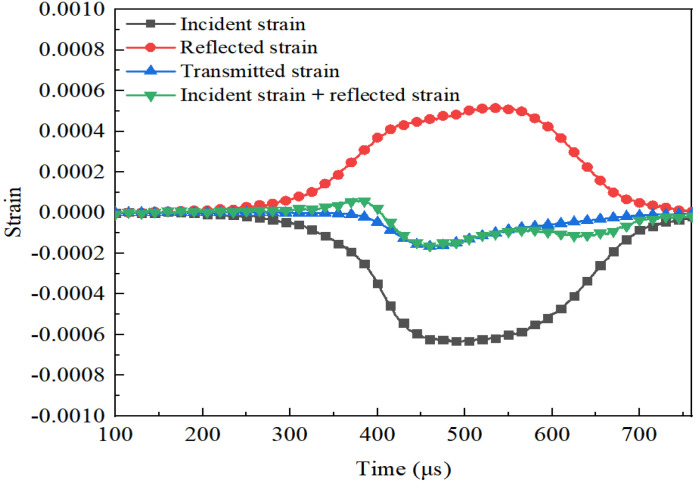
Stress equilibrium for SHPB testing.

The 100 mm-diameter SHPB testing system used in this research is shown in Figure 5. All the bars are made of high-strength alloy steel, with a density of 7850 kg/m^3^ and an elastic modulus of 210 GPa. The sensitivity coefficients of the semiconductor strain gauge and resistance strain gauge used in this test are 105 and 2.2, respectively. To satisfy the uniform stress hypothesis in the SHPB test of 3DPCM, some measures were taken to improve the validity of test data. A moderate amount of Vaseline was smeared on the bar-specimen interfaces to reduce the friction between the specimen and the end-face of the bars. To reduce the effects of dispersion, a rubber disk used as a pulse shaper was installed in front of the incident bar, measuring 30 mm in diameter and 2 mm thick. Figure 6 presents the stress equilibrium for dynamic compression test: the curve after superposition of the incident and reflected waves was almost identical with the transmitted wave, showing that the dynamic stress equilibrium at both ends of the specimen was reached. In this test, the impact pressure was 0.1 MPa and the corresponding strain rate was approximately 120 s^−1^. The SHPB specimens with the same S/B were divided into four groups: mold cast specimens, and three groups of printed specimens (X, Y, and Z-directions); each group included three identical specimens, and the result of the corresponding group was the average value of three test specimens.

On the basis of one-dimensional wave theory [32], the incident pulse $\varepsilon_{i}(t)$, reflected pulse $\varepsilon_{r}(t)$ and transmitted pulse $\varepsilon_{t}(t)$ measured by strain gauges are used for calculating the dynamic compressive strength σ(t), strain ε(t) and strain rate $\overset{•}{\varepsilon }(t)$ histories as follows:

(2) $$\left\{ \begin{array}{l} \sigma (t)=EA\varepsilon_{t}(t)/A_{s}\\ \varepsilon (t)=-2C\int_{0}^{t}\varepsilon_{r}(t)dt/L_{s}\\ \overset{•}{\varepsilon }(t)=-2C\varepsilon_{r}(t)/L_{s} \end{array}\right.$$

where *E*, *A,* and *C* are defined as the elastic modulus, cross-sectional area, and longitudinal wave velocity of bars, respectively; *A_s_* and *L_s_* are the cross-sectional area and length of the SHPB test specimen, respectively.

## 3. Results and Discussion

### 3.1. Fractal Dimension of Failed 3DPCM Specimens

**Figure 7:**
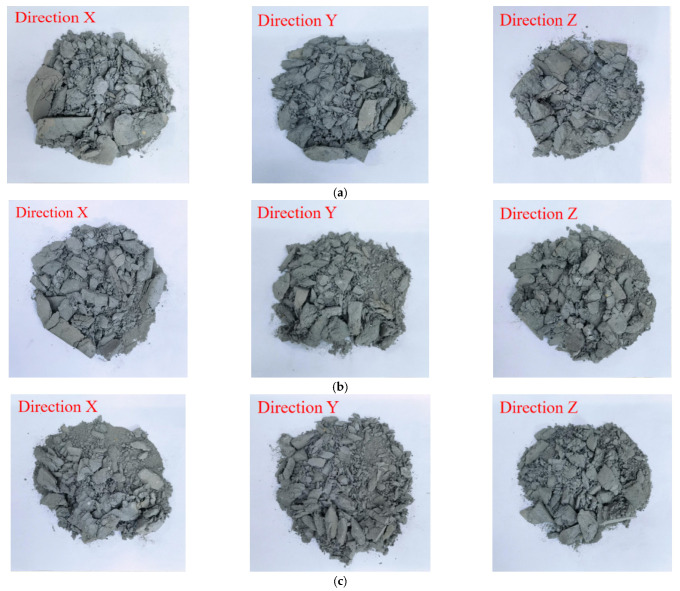
Failure modes of printed specimens in different printing directions. (**a**) S/B = 0.8 (**b**) S/B = 1.1 (**c**) S/B = 1.4.

**Figure 8:**
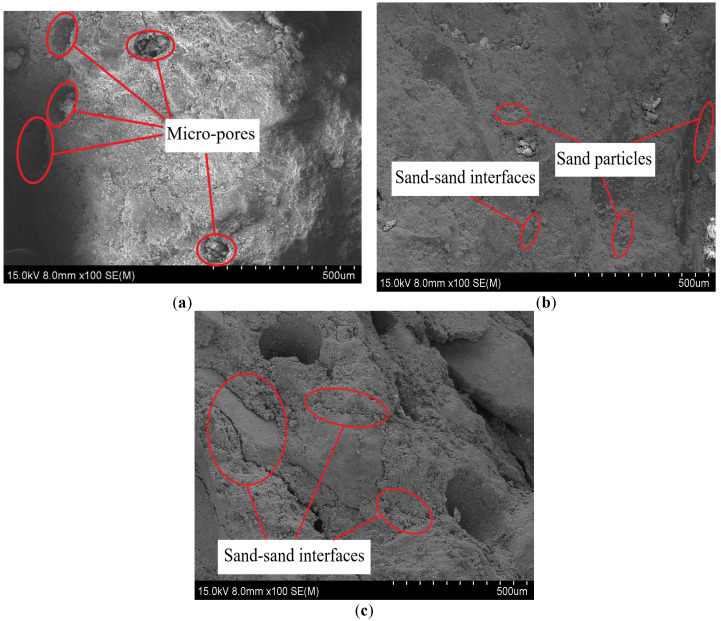
SEM images of the printed sample with different sand to binder ratios (100×). (**a**) S/B = 0.8 (**b**) S/B = 1.1 (**c**) S/B = 1.4.

**Figure 9:**
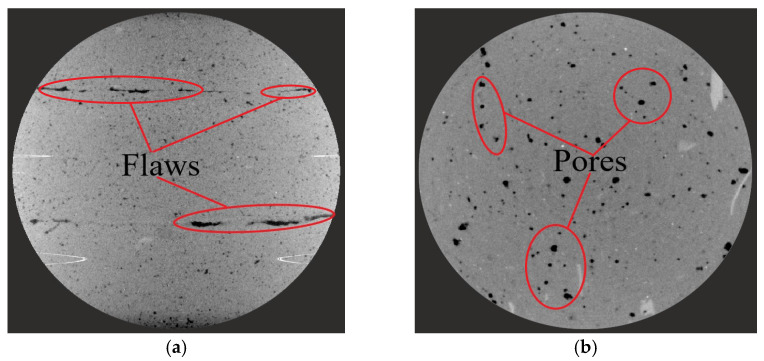

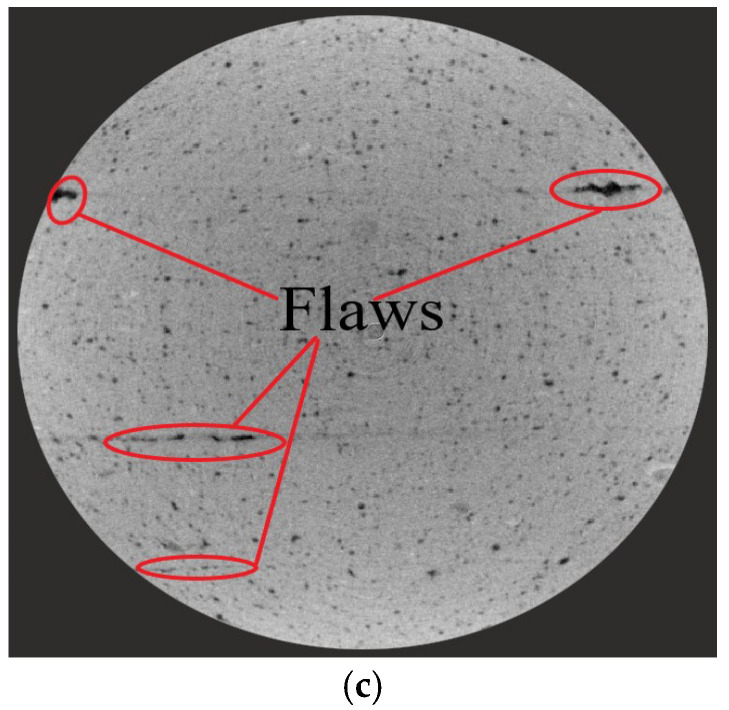
CT scanning images of the sample in different printing directions (1.1×). (**a**) Printing X-direction (**b**) Printing Y-direction (**c**) Printing Z-direction.

The change of S/B was achieved by keeping the amount of binding material constant and changing the addition of sand. Figure 7 shows that the different printing directions and sand to binder ratios exert influences on the size and number of broken fragments, leading to the different failure modes of printed mortars. With the increase of the S/B (when the sand content increases), it induces the formation of more sand–sand interfaces formed inside printed samples (Figure 8). The internal cracks and defects formed by the sand–sand interfaces can easily expand and connect, which makes the printed samples prone to break into pieces of different sizes and shapes. Therefore, the large-size fragments (>26.5 mm) in the same printing direction are decreased and gradually transformed into medium to small-size fragments (<2.36 mm) with increases in S/B. Additionally, for the same case of S/B, the size of broken fragments in the printing Y-direction is significantly decreased compared to that in the other two printing directions. This result can be explained by the different shapes of defects in different printing directions. Figure 9 demonstrates that the main defects in the printing Y-direction are circular pores, which are slender cracks in the printing X and Z-directions. Under the same impact, micro cracks are formed by the expansion and connection of pores, which can eventually penetrate the printed sample after their propagation and lead to macroscopic destruction of the sample. Compared with pore defects, the penetration of crack defects causes the earlier failure of samples, and many other internal defects have no time to expand or connect into new cracks, resulting in the smaller crack density and larger broken fragments.

**Figure 10:**
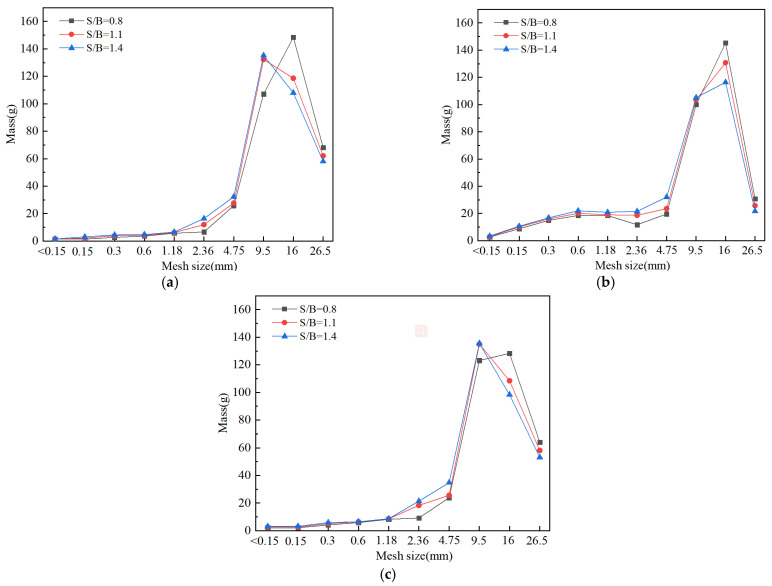
Various sizes of the fragments in different printing directions. (**a**) Printing X-direction (**b**) Printing Y-direction (**c**) Printing Z-direction.

The particle size distribution of destructed 3DPCM is obtained by sieving, and the results in different printing directions are demonstrated in Figure 10.

The fractal dimension is usually adopted to quantify the extent of impact crushing. The relationship between the partial mass and total mass can be obtained with reference to the functional model [33] and the mass-frequency relationship [34]. Then, invoking the mass-granularity method [25], the fractal dimension *D_F_* is given by:

(3) $$\ln(M_{r}/M_{t})=(3-D_{F})\ln(r/r_{\max})$$

where *M_r_* and *M_t_* are the accumulated mass of fragments with a particle size less than *r* and the total mass of fragments, respectively; *r* and *r_max_* are the particle size of fragments and the maximum particle size of fragments, respectively.

**Figure 11:**
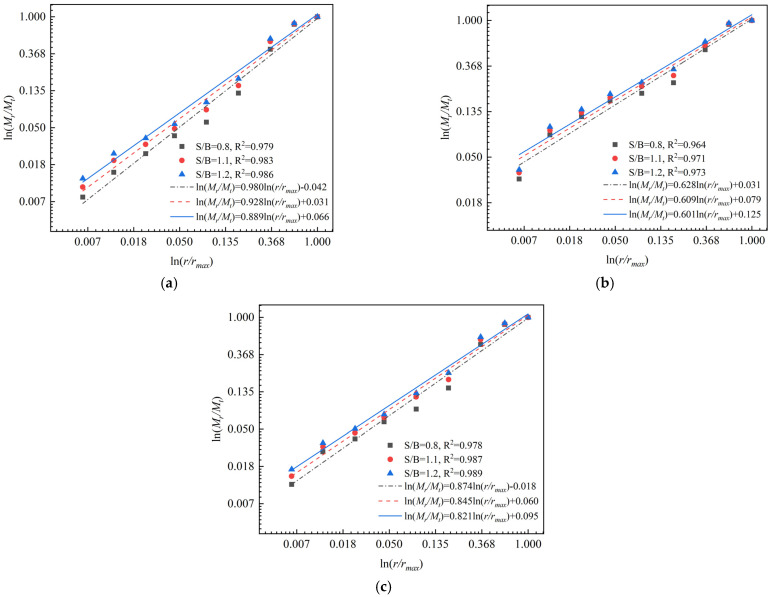
ln*(M_r_/M_t_)*–ln*(r/r_max_)* plots for failed 3DPCM in different printing directions. (**a**) Printing X-direction (**b**) Printing Y-direction (**c**) Printing Z-direction.

As (3–*D_F_*) is the slope of the ln*(M_r_/M_t_)–*ln*(r/r_max_)* lines, *D_F_* can be obtained therefrom. According to Equation (3) and sieving results of particle size distribution, the relationship between the the relative mass and the relative size of the destructed 3DPCM in different printing directions is illustrated in Figure 11.

As presented in Figure 11, the correlation coefficients of straight fitting lines are all above 0.96, indicating the broken fragments in the same printing direction exhibit self-similarity and fractality. The defects randomly distributed inside internal structure have significant self-similarity [28]. Due to the evolution, connection, and propagation of micro-pores, micro-cracks, and printing defects randomly distributed inside 3DPCM, the printed specimens are damaged under impact loading. Therefore, the broken fragments of destructed 3DPCM in the same printing direction show self-similarity characteristics.

**Figure 12:**
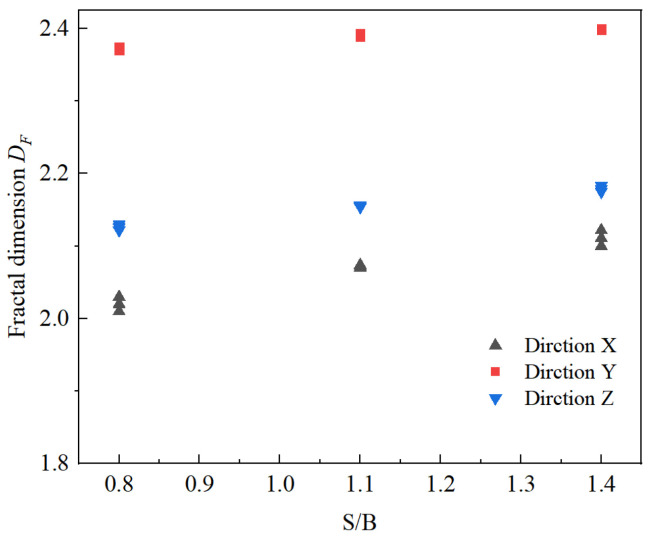
Relationship between fractal dimension and S/B.

The fractal dimension *D_F_* illustrated in Figure 12 increases gradually as S/B increases from 0.8 to 1.4. Compared with the case of S/B = 0.8, the average fractal dimension at S/B = 1.4 in the printing X, Y, and Z-directions is increased by 4.50%, 1.14%, and 2.49%, respectively. In the same printing direction, increasing sand–sand interfaces are formed inside the printed mortars with the increase of S/B, which are prone to slip and develop into cracks. Therefore, the crushing of 3DPCM becomes more serious as S/B increases, and the corresponding fractal dimension gradually increases. In addition, at the same S/B, the structural anisotropy of the printed samples leads to the differences in strength in the different printing directions [35]. The differences in strength under the same impact pressure eventually lead to the differences in the extent and severity of crushing, as evinced by the differences in fractal dimension. When sand to binder ratios are 0.8, 1.1, or 1.4, the average fractal dimensions obtained in the Y printing direction are respectively increased by 17.43%, 15.40%, and 13.64% compared to that in the X printing direction. It is found that the variation of the fractal dimension is only slightly influenced by S/B, but is significantly affected by the printing direction.

### 3.2. Evaluation of Compactness through UPV Testing

**Figure 13:**
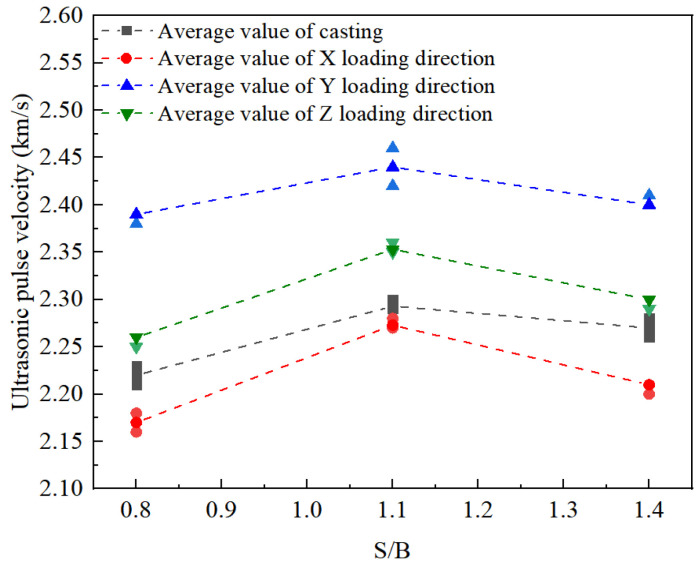
UPV of specimens with different S/B ratios.

Ultrasonic pulse testing is a non-destructive testing method; the slower the UPV, the more numerous the internal defects and the lower the density [31]. When S/B is in the range of 0.8 to 1.4, the change in UPV of the printed mortar in different printing directions is illustrated in Figure 13. At the same S/B, the density in the printing Y-direction is the highest with the fastest UPV, but that in the printing X-direction is lowest with the slowest UPV. In the same printing direction, the UPV continues increasing until S/B reaches 1.1, and then gradually decreases, suggesting that the density of the printed mortars first increases, then decreases with increasing S/B. Moreover, under the same change of S/B, the change of ultrasonic pulse velocity in the printing direction Z is smaller than that in the printing direction X, but larger than in printing direction Y. Correspondingly, the change in density in the printing X-direction is the largest, but that in printing Y-direction is the smallest.

### 3.3. Dynamic Compressive Strength

#### 3.3.1. Dynamic Stress-Strain Curves of 3DPCM

**Figure 14:**
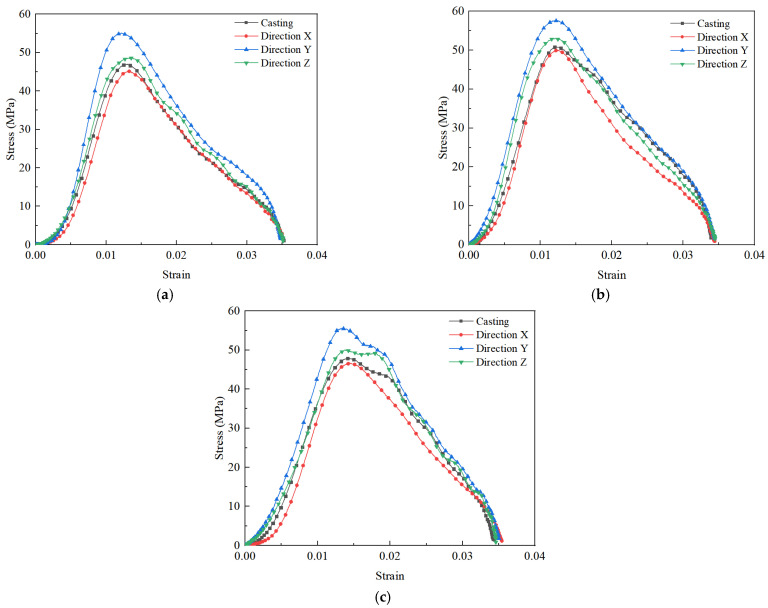
Dynamic stress–strain curves of 3DPCM. (**a**) S/B = 0.8 (**b**) S/B = 1.1 (**c**) S/B = 1.4.

In this work, the impact compression test specimens processed from the printing X, Y, and Z-directions of 3DPCM are denoted by DX, DY, and DZ, respectively. In addition, the impact compression specimens processed from mold cast cement mortar are denoted by DC. Figure 14 illustrates the dynamic compressive stress–strain curves of 3DPCM at various sand to binder ratios and printing directions. The stress–strain curve generally consists of three phases: (1) the elastic stage, the dynamic stress approximately linearly increases with the increase in strain; (2) the yield stage, the stress–strain curves begin to be non-linear and the slope thereof decreases before the stress reaches its largest value as the strain increases, corresponding to the development of bond cracks; (3) the failure stage, the stress decreases rapidly, under a slow increase in the strain, showing the load-bearing capacity of specimens decreases gradually. Furthermore, the dynamic compressive strength and ultimate strain are defined as the peak stress and final strain on the stress–strain curves, respectively. The dynamic compressive strength varies with the printing direction and S/B, however, ultimate strains display a convergence regardless of the change of printing direction and S/B, and become similar at 0.035. Therefore, it is evident that the dynamic compressive strength of 3DPCM is affected by the printing direction and S/B, but the ultimate strain remains unaffected.

#### 3.3.2. Variations of Dynamic Compressive Strength versus S/B and Printing Directions

**Figure 15:**
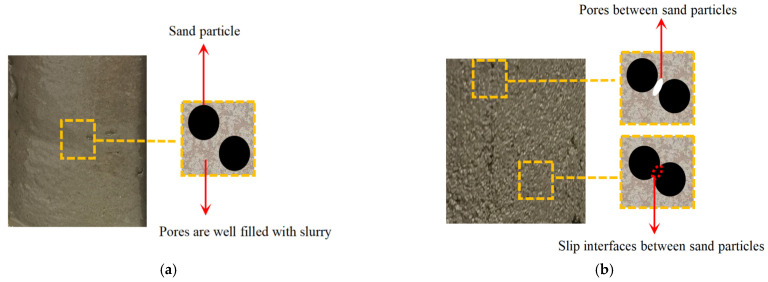
Schema of sand distribution in the internal structure. (**a**) With the appropriate amount of sand (**b**) With excess addition of sand.

At S/B is 0.8 (Figure 14a), and the dynamic compressive strengths of DC, DX, DY and DZ are 47.01 MPa, 45.16 MPa, 55.07 MPa and 48.59 MPa, respectively. The dynamic compressive strengths between 3D printed cement mortars in different printing directions and mold cast cement mortar are ranked (in ascending order) as follows: DY, DZ, DC, then DX, demonstrating the anisotropic dynamic compressive strength of 3DPCM. Similar results are obtained at S/B = 1.1 (Figure 14b) and S/B = 1.4 (Figure 14c). There exists weak bonding between filaments caused by air voids and longitudinal defects between adjacent filaments formed during the printing process [14]. As a result, the air voids and longitudinal defects form weak bonding surfaces between strip-like filaments. The more defects there are, the more numerous are the weak interfaces formed, causing the specimen to be more easily broken under impact, thus lowering the dynamic compressive strength. Moreover, the dynamic compressive strength is not only dependent strongly on the printing direction, but is also affected by S/B. When S/B increased from 0.8 to 1.1, some air voids are gradually filled by the added sand particles, so the strength is increased because of the increased density [21]. Moreover, sand particles play a major supporting role in the internal structure of printed cement mortars and adding a certain amount of sand can improve the strength [36]. When S/B reaches 1.4, the presence of excessive sand particles leads to a reduction of the slurry layer thickness, resulting in the decline of the bonding force of sand particle–cementitious gel interfaces [37]. In addition, as shown in Figure 15, pores and slip interfaces between sand particles are formed within the printed cement mortars with the excess addition of sand [38], resulting in the decrease of dynamic compressive strength in the same printing direction.

#### 3.3.3. Anisotropy of 3DPCM with Different Sand to Binder Ratios

The anisotropic coefficient *I_d_* is introduced to quantify the anisotropy of dynamic compressive strength, which can be represented by the Equation (4) [14].

(4) $$I_{d}=\sqrt{{\left(f_{X}-f_{C}\right)}^{2}+{\left(f_{Y}-f_{C}\right)}^{2}+{\left(f_{Z}-f_{C}\right)}^{2}}/f_{C}$$

where *f_X_, f_Y_, f_Z_,* and *f_C_* represent the dynamic compressive strengths of DX, DY, DZ, and DC, respectively.

Since the mold cast specimen is considered homogeneous, the calculated anisotropic coefficient is 0, however, the difference is that the printed specimen is considered to have a heterogeneous meso-structure, thus the larger the anisotropic coefficient, the greater the anisotropy.

**Figure 16:**
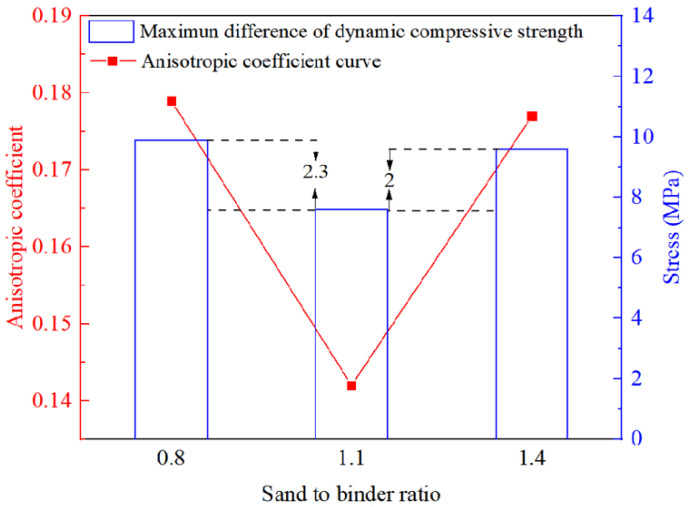
Maximum difference of compressive strength and anisotropic coefficient with different sand to binder ratios.

As shown in Figure 16, the anisotropic coefficient of 3DPCM decreases from 0.179 to 0.142, corresponding to a decrease of 20.67% as S/B increases from 0.8 to 1.1. When S/B reaches 1.4, the anisotropic coefficient increases to 0.157, which shows an increase of 10.56% compared with that at S/B = 1.1. This phenomenon is caused by the differences in defects (and thus density variation) in different printing directions. According to the data in Section 3.2, the changes in density of DX and DY are the largest and the smallest, respectively under the same change in S/B. Appropriate addition of sand can improve the density of specimens formed in different printing directions to different degrees and reduce the differences in defects therein. Consequently, the maximum difference in the dynamic compressive strength of 3DPCM in different printing directions decreases by 2.3 MPa when S/B is increased from 0.8 to 1.1, and the anisotropic coefficient decreases accordingly. Moreover, the difference in density between different printing directions increases with the addition of excess sand, leading to the increase of anisotropy, which is also reflected in the maximum difference in strength being increased by 2.0 MPa as S/B is increased to 1.4.

### 3.4. Relationship of Dynamic Compressive Strength to Fractal Dimension

**Figure 17:**
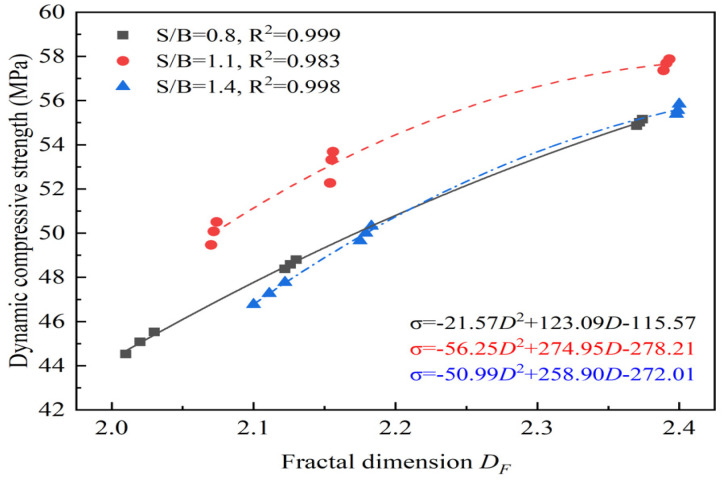
Correlation of dynamic compressive strength with fractal dimension.

For the same case of S/B, the dynamic compressive strength and fractal dimension of 3DPCM in three printing directions are non-linearly fitted, as shown in Figure 17. When the fractal dimension increases from 2.0 to 2.4, there is a quadratic relationship between dynamic compressive strength and fractal dimension of 3DPCM in different printing directions. From the perspective of fractures mechanics, the greater the strain energy, the more micro-cracks and flaws are activated, leading to an increase in the number of broken fragments and energy dissipation [34], and the increased energy dissipation improves the dynamic compressive strength [28]. As a result, the greater the dynamic compressive strength in the printing direction, the larger the fractal dimension, and the dynamic compressive strength with the same S/B is positively correlated with the fractal dimension.

**Figure 18:**
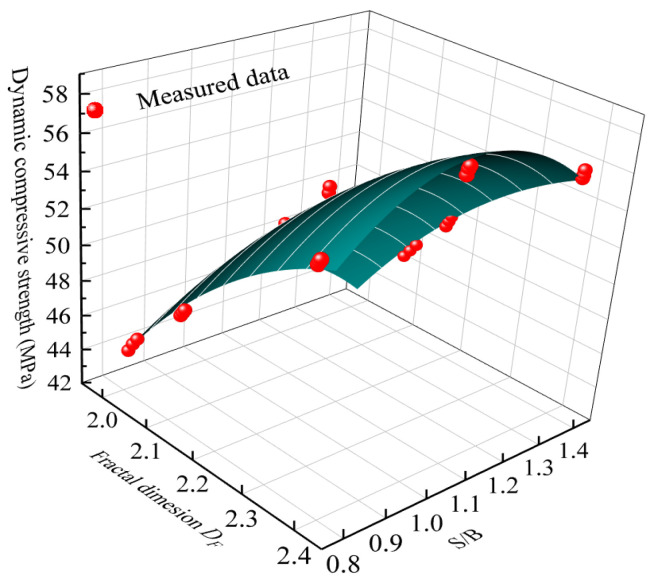
Relationship of dynamic compressive strength–fractal dimension–S/B.

Since the dynamic compressive strength and fractal dimension are both affected by S/B, the relationship of dynamic compressive strength and fractal dimension with the change of S/B can be determined from Figure 18, as expressed as Equation (5).

(5) $$\sigma =195.04D_{F}+62.46(S/B)-39.43D_{F}^{2}-35.71(S/B{)}^{2}+6.92D_{F}(S/B)-226.35$$

### 3.5. Variations of Energy Dissipation Density versus S/B and Printing Directions

According to the law of thermodynamics, the physical change process of materials is essentially the result of energy conversion, and the macroscopic failure of materials is accompanied by energy absorption, dissipation, and release [39]. The total absorbed energy *W_s_* can be obtained indirectly from the difference of incident energy *W_i_*, reflected energy *W_r_* and transmitted energy *W_t_*. In addition, different types of energy *W_I_* can be determined by the corresponding pulse *ε_I_* measured in the SHPB test [40]. The governing expression is:

(6) $$\left\{ \begin{array}{l} W_{I}=EAC\int \varepsilon_{I}^{2}(t)dt,\;I=i,r,t\\ W_{s}=W_{i}-W_{r}-W_{t} \end{array}\right.$$

The energy dissipation density *U* reflects the energy consumed by the material per unit volume and the dissipated energy *W_d_* is approximately equal to the total absorbed energy *W_s_* when the loading rate is not very high [41]. Accordingly:

(7) $$U=\frac{W_{d}}{A_{S}L_{S}}=\frac{W_{S}}{A_{S}L_{S}}$$

**Figure 19:**
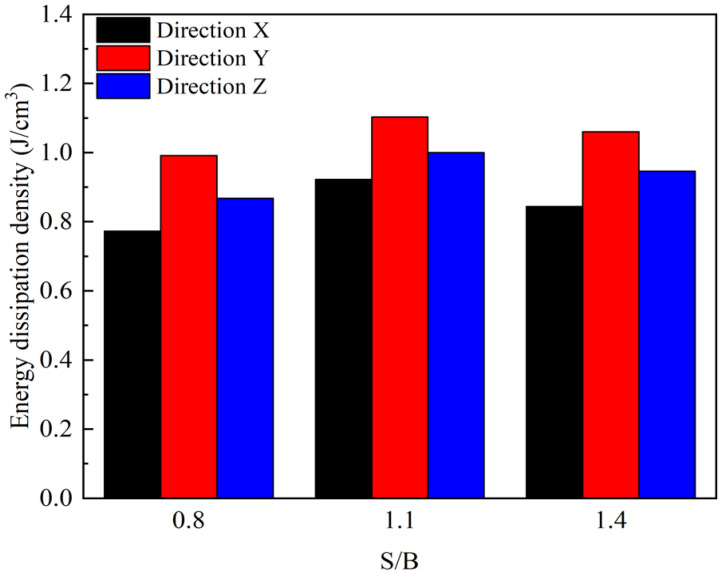
Energy dissipation density of 3DPCM with different sand to binder ratios.

Before S/B reaches the optimal value of 1.1, there is an improvement in the structural compactness of 3DPCM due to the filling of original pores, and many new micro-cracks are generated when the printed mortar is exposed to the same impact. Compared with the original crack propagation, the formation of new cracks requires much more dissipated energy [28]. Consequently, when S/B is between 0.8 and 1.1, the energy dissipation densities of DX, DY, and DZ are increased by 28.20%, 19.63%, and 25.59%, respectively, as shown in Figure 19, however, when S/B exceeds the optimal value, many sand–sand interfaces are formed inside the internal structure. Since the sand–sand interfaces have poorer stability compared with the sand–cementitious gel interfaces, which readily slips and damages the structure [38]. Thus, the rapid expansion of the micro-cracks in the sand–sand interfaces leads to the ultimate failure of 3DPCM, leaving no time for new cracks to form elsewhere. As a result, the energy dissipation density declines with the further increase of S/B, which in the printing X, Y, and Z-directions decreases by 12.41%, 9.34%, and 10.75%, respectively. 

**Figure 20:**
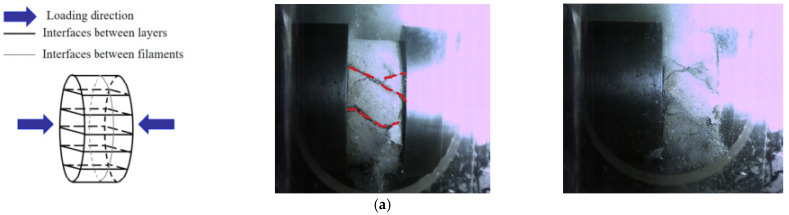

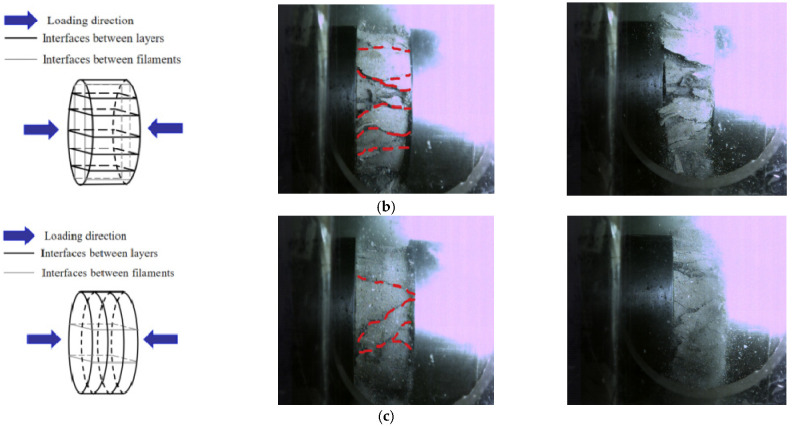
Interface distribution and main cracks of 3DPCM in different printing directions. (**a**) Printing X-direction (**b**) Printing Y-direction (**c**) Printing Z-direction.

In addition, the energy dissipation density of different printing directions is obviously different: at a given S/B, the energy dissipation density of DY is the largest, while that of DX is smallest. This is mainly dependent on the difference of the internal structural in different printing directions. For 3D printed specimens DX and DZ, it can be considered as having been formed by the bonding of short beams. Under impact, there are inclined shearing cracks generated along the weak interface (Figure 20a,c). When the shear failure occurs along the weak interface in the specimen, the dynamic compressive strength of the material is not fully mobilized [32]. As the main cracks (red dotted lines in Figure 20) propagate through the specimen, other internal micro-cracks have little time to expand or connect along the weak interface and it is difficult for new main cracks to form. Compared with DZ, DX is more prone to instability and failure due to the lower strength, resulting in the reduced formation of main cracks after impact. Different from DX and DZ, the printed filaments of DY are parallel to the direction of the applied load. In this case, the printed mortar is in fact subjected to dynamic stress by evenly distributed short columns, and most of the main cracks are longitudinal cracks formed parallel to the weak surface, as shown in Figure 20b. Due to the better compressive resistance of short columns subject to a uniform load distribution, the compressive strength of the specimen is improved [14]. When DY is destroyed due to the failure of the short columns, many new micro-cracks are formed: these then expand and interconnect, hence the greater number of main cracks in DY than in DX and DZ. Since the formation of main cracks requires energy absorption [42], the larger the number of main cracks, the more energy is absorbed, so the energy dissipation density of DY under the same impact load is greater.

### 3.6. Relationship of Energy Dissipation Density to Fractal Dimension

**Figure 21:**
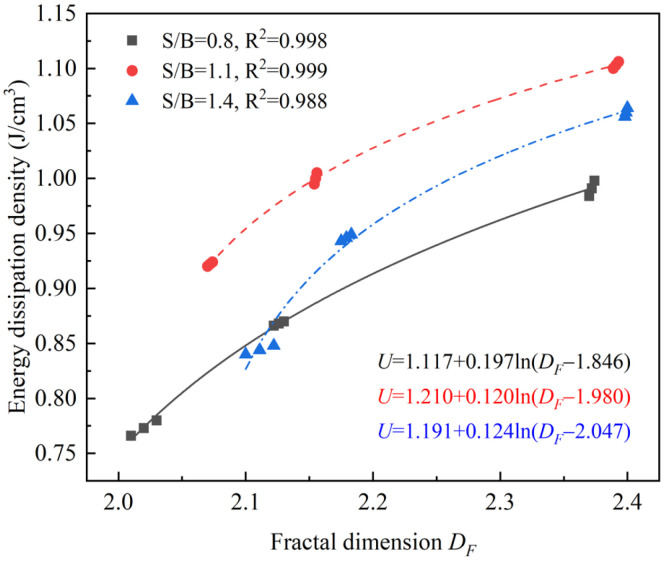
Correlation of energy dissipation density with fractal dimension.

As illustrated in Figure 21, under the same S/B, the relationship between the energy dissipation density and fractal dimension in different printing directions can be obtained by non-linear fitting: the energy dissipation density in different printing directions has a logarithmic relationship with fractal dimension. From the energy point of view, a certain amount of energy was absorbed by the printed specimen under impact, which is instantly dissipated upon the instability and failure of the specimen and drives the propagation, connection and extension of micro-cracks [39]. In other words, the energy-driven instability process caused the macroscopic failure of printed specimens [28], indicating that the larger the energy dissipated, the more numerous the cracks, the more severe the damage to the specimens, and the larger the corresponding fractal dimension. The results that energy dissipation density is the largest in the printing Y-direction and the smallest in the printing X-direction conforms well with the measured results showing that the fractal dimension is the highest in the printing Y-direction and the lowest in the X-direction.

**Figure 22:**
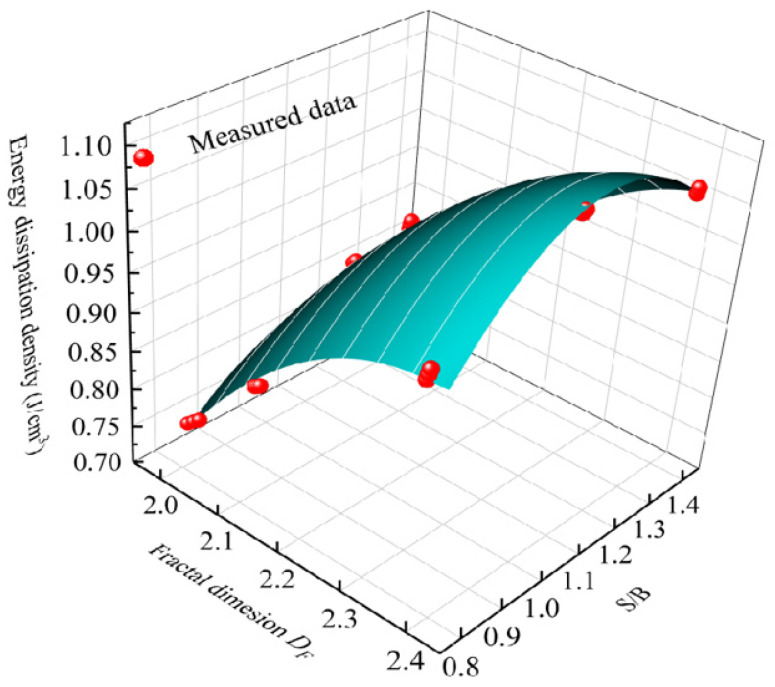
Relationship of energy dissipation density to fractal dimension–S/B.

Through the previous analysis, the energy dissipation density of 3DPCM under impact loading is dependent on S/B and inherently related to the fractal dimension. The relationship of energy dissipation density to S/B and fractal dimension can be obtained by fitting the data in Figure 22; the relevant expression is

(8) $$U=7.84D_{F}+1.31(S/B)-1.65D_{F}^{2}-1.04(S/B{)}^{2}+0.46D_{F}(S/B)-8.73$$

### 3.7. Manufacturing of the 3D Printed Barracks Model

**Figure 23:**
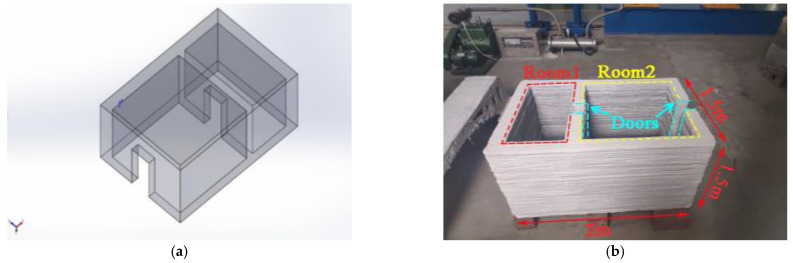
3D printed barracks model. (**a**) Model structure design (**b**) Printed model.

Among the printable cement mortars prepared in the course of the present research, the mortar mix with S/B = 1.1 was selected for the 3D printing of the barracks model because of its optimal dynamic mechanical properties. As presented in Figure 23, the model measuring 2 m (L) × 1.5 m (W) × 1.5 m (H) consists of two spaces and doors. The entire printing process was finished (without using formwork) within 2 h at a print-head speed of 9 m/min. Moreover, the printed model will be used for future explosion impact experiments to explore the corresponding damage characteristics of a 3D printed structure.

## 4. Conclusions

In this study, SHPB tests on 3DPCM with different sand to binder ratios were performed in three printing directions. The fractal feature, dynamic compressive behavior, and energy dissipation characteristics of destructed 3DPCM were investigated. Moreover, the effects of S/B on the anisotropy of the dynamic compressive strength of 3DPCM were evaluated. Main conclusions can be drawn from the current study.

1. The dynamic compressive strength of 3DPCM in the different printing directions has various values even at the same S/B, showing the anisotropic characteristics of the printed mortar. The relationship between the dynamic compressive strength of 3DPCM in different printing directions and the compressive strength of mold cast cement mortar is ranked (in descending order) as follows: DY, DZ, DC, then DX. Additionally, the internal defects are well filled so that the printed specimen is more dense when S/B is 1.1, and the dynamic compressive strength in the same printing direction reaches its maximum value.
2. For the same change in the value of S/B, the difference in density changes caused in different printing directions causes various variations of the anisotropic coefficient. When S/B increases from 0.8 to 1.4, the anisotropic coefficent of 3DPCM first decreases by 20.67%, then increases by 10.56%, indicating the anisotropy of the dynamic compressive strength first becomes mitigated, and then increases.
3. The difference of the internal structure in different printing directions can result in significantly different energy dissipation densities, which directly reflects various numbers of main-cracks formed during the failure process. At the same S/B, the energy dissipation density of DZ is larger than that of DX, but smaller than DY. Moreover, the energy dissipation density in the same printing direction first increases, then declines as S/B is increased from 0.8 to 1.4.
4. At a given S/B, the fractal dimension of DY is largest among the three printing directions and the fractal dimension of 3DPCM gradually increases with increasing S/B. Compared with S/B, the printing direction has a more significant influence on the fractal dimension. The higher the dynamic compressive strength in the same printing direction, the greater the energy dissipation density, the more severe the impact crushing, and the larger the fractal dimension. When the fractal dimension is in the range of 2.0 to 2.4, it has a quadratic relationship with the dynamic compressive strength and a logarithmic relationship with the energy dissipation density.

## Figures and Tables

**Table 1:** Research into the interlayer bond strength of 3D printed cementitious materials.

Reference	Study Findings
Wolf et al. [15]	The bond strength of 3D printed concrete was reduced significantly when specimens were exposed to dry curing conditions, resulting in the decrease of compressive strength.
Sanjayan et al. [16]	The decrease in the surface moisture content with the increasing print-time interval caused the decline of interface bond strength between layers.
Panda et al. [17]	The low yield strength material could be well mixed with the previous layer to form a good bond strength; reducing the nozzle height improved interface bond strength.

**Table 2:** The dynamic properties of cementitious materials as evinced by SHPB tests.

Reference	Research Contents
Wang et al. [20]	The dynamical compressive properties of fiber reinforced high strength concrete (FRHSC)
Ma et al. [21]	The dynamic behavior and the absorbed energy of basalt fiber reinforced cement soil
Li et al. [22]	The dynamic compressive properties of light-weight geopolymer composites

**Table 3:** Particle size distribution of river sand.

Diameter (mm)	≥2.5	1.18–2.5	0.63–1.18	0.315–0.63	0.16–0.315	<0.16
Percentage by mass (%)	0.40	1.52	2.30	50.48	41.38	3.92

**Table 4:** The weight ratio of raw materials and printability test results.

Cement	Silica Fume	Fly Ash	Sand	Water	Superplasticizer	Thicker Agent	Workability (mm)	Buildability (mm)
1	0.02	0.33	0.8	0.34	0.001	0.0005	190	77
1	0.02	0.33	1.1	0.34	0.001	0.0005	172	79
1	0.02	0.33	1.4	0.34	0.001	0.0005	156	80

## Data Availability

The data presented in this study are available on request from the corresponding author.
